# Fiber-based vegetarian score and the risk of incident dementia among elderly Chinese adults: a prospective cohort study

**DOI:** 10.3389/fnut.2026.1819946

**Published:** 2026-05-25

**Authors:** Wanlin Zhou, Dongdong Yang, Keyun Guo, Ling Quan, Zhuoling Li, Xingyu Wang, Huilian Cao, Wen Xiao

**Affiliations:** 1 Hospital of Chengdu University of Traditional Chinese Medicine, Chengdu, China; 2 Chengdu University of Traditional Chinese Medicine, Chengdu, China

**Keywords:** cognitive function, cohort study, dementia, elderly people, vegetarian diet

## Abstract

**Background:**

With the acceleration of population aging, dementia has become a major public health challenge. Although vegetarian diets have been proven to be related to cognitive health in Western populations, there is a lack of longitudinal research evidence in the elderly population in China. This study aims to use data from the China Longitudinal Healthy Longevity Survey (CLHLS) to prospectively explore the association between vegetarian dietary patterns and the risk of dementia.

**Methods:**

This study included 1,948 elderly individuals aged ≥ 65 years at baseline and without dementia in the CLHLS 2008–2018 cohort. The frequency of intake of 10 types of foods was assessed using a food frequency questionnaire, and a vegetarian dietary score was constructed based on the intake frequency of vegetarian foods (grains, vegetables, fruits, nuts, fungi). During follow-ups in 2010, 2011, 2014, and 2018, dementia onset events were identified through Chinese version of the Mini-Mental State Examination (CMMSE), activities of daily living ability, and self-reported physician diagnoses. The Cox proportional hazards model was used to estimate hazard ratios (HR) and 95% confidence intervals (CI), with adjustment for confounding variables including sociodemographic characteristics, lifestyle, and health status. Subgroup analyses and sensitivity analyses were conducted to evaluate effect modification and the robustness of the results.

**Results:**

During the 10-year follow-up period, a total of 53 incident dementia cases (2.7%) were documented. After multivariate adjustment, each 1-point increase in the vegetarian dietary score was associated with a 17% reduction in dementia risk (HR = 0.83, 95% CI: 0.70–0.98, *p* = 0.031). Compared with participants in the lowest tertile of vegetarian score, those in the intermediate tertile (HR = 0.49, 95% CI: 0.26–0.93, *p* = 0.028) and the highest tertile (HR = 0.50, 95% CI: 0.23–0.99, *p* = 0.048) had significantly lower risks of dementia. Subgroup analyses revealed that the protective effect of a vegetarian diet was more pronounced among participants aged < 80 years (HR = 0.62, 95% CI: 0.48–0.80), non-drinkers (HR = 0.81, 95% CI: 0.67–0.97), those without formal education (HR = 0.79, 95% CI: 0.63–0.98), those engaged in physical labor (HR = 0.66, 95% CI: 0.49–0.89), and those married/cohabiting (HR = 0.70, 95% CI: 0.54–0.90). After excluding cases with early onset during follow-up and extreme age values, sensitivity analyses confirmed the robustness of the findings.

**Conclusion:**

A vegetarian dietary pattern is significantly associated with a lower risk of dementia in Chinese elderly individuals, and this protective effect varies across demographic and lifestyle subgroups. This study supports the inclusion of vegetarian diets patterns in primary prevention strategies for dementia and highlights their particular application value in specific vulnerable populations.

## Introduction

Against the backdrop of rapid global population aging, dementia has emerged as a critical public health challenge, characterized by its high incidence, irreversible progression, and substantial socioeconomic burden (1). Epidemiological evidence indicates that the global burden of dementia has accelerated markedly since 2019, with age serving as the predominant non-modifiable risk factor; the risk of developing dementia increases exponentially after age 60 (2). In China, data from 2021 reveal that the prevalence and mortality rates of dementia reached 1,194.2 per 100,000 population and 34.6 per 100,000, respectively (3), with projections indicating that the prevalence among Chinese older adults will continue to increase in the coming decades (4). As a neurodegenerative disorder characterized by progressive cognitive decline, dementia not only severely impairs patients’ daily functioning and quality of life but also places immense strain on family caregiving systems and healthcare resources (5). Although no curative treatments currently exist for dementia, a growing body of evidence suggests that effective management of modifiable risk factors could prevent or delay the onset of more than one-third of cases (6).

Current evidence indicates that dementia onset results from the complex interplay between genetic and environmental factors. A range of modifiable environmental factors have been implicated in dementia risk, including lower educational attainment, physical inactivity, social isolation, and unhealthy dietary patterns (7, 8). Among these, accumulating evidence from prospective cohort studies has demonstrated that adherence to healthy dietary patterns—such as the Mediterranean diet, DASH diet, and plant-based diets—is associated with slower cognitive decline and reduced risk of incident dementia (9, 10). Consequently, dietary patterns represent a key modifiable determinant and offer a crucial target for dementia prevention.

The biological plausibility of plant-based dietary patterns in neuroprotection is supported by multiple interconnected mechanisms. Specifically, dietary fiber—a hallmark component of plant-based foods—is fermented by the gut microbiota to produce short-chain fatty acids (SCFAs) (11). SCFAs have been shown to regulate neuroinflammation, strengthen gut-brain barrier integrity, and reduce oxidative stress, all of which are directly implicated in the pathogenesis of dementia (12). In parallel, plant-based foods provide abundant polyphenols and vitamins with potent antioxidant and anti-inflammatory properties. Consequently, plant-based dietary patterns—particularly those rich in dietary fiber—have garnered considerable attention in neuroprotection research.

However, existing research has primarily focused on Western populations or broadly defined plant-based dietary patterns. For instance, a UK Biobank study of 107,785 older adults reported that a higher carbohydrate-to-fiber ratio was associated with a 7% increased risk of dementia per standard deviation, accompanied by reduced entorhinal and frontal cortical thickness (13). Likewise, data from the NHANES showed that the protective association between dietary fiber intake and cognitive function was partially mediated by anti-inflammatory markers, with a mediation proportion of 17.88% (14). Together, these findings indicate that dietary fiber may serve as a key nutritional component for dementia prevention, acting through gut-brain axis and anti-inflammatory pathways.

A recent cross-sectional study by Xian et al. (15) using data from the 2018 wave of the CLHLS examined the association between plant-based dietary patterns and dementia. The study reported that a healthy plant-based dietary index was associated with lower odds of dementia (OR = 0.976, 95% CI: 0.963–0.990) (15). However, the dietary indices employed by Xian et al. (PDI, hPDI, uPDI) broadly aggregated all plant-derived foods without distinguishing foods based on their dietary fiber content or their distinct neuroprotective mechanisms. Consequently, these indices do not directly reflect dietary fiber intake. To address this gap, the present study constructed a fiber-based vegetarian score, which selectively includes five fiber-rich food categories (grains, vegetables, fruits, nuts, and fungi). Using longitudinal data from the CLHLS (2008–2018 waves) and Cox proportional hazards regression models, we prospectively examined the association between this fiber-focused scoring system and incident dementia among Chinese older adults.

Importantly, the present study does not include exclusively vegetarian participants (i.e., those who completely abstain from animal products). Rather, we operationalize the consumption frequency of fiber-rich plant foods as a continuous fiber-based vegetarian score, which serves as a proxy for daily dietary fiber intake. This terminology aligns with current nutritional epidemiology practices and avoids misleading connotations of strict vegetarianism. Accordingly, we hypothesized that higher adherence to the fiber-based vegetarian score would be associated with a lower risk of incident dementia. This study aims to provide evidence-based dietary guidance for dementia prevention in this population and to strengthen the evidence base supporting the modifiability of dementia risk through nutritional interventions with a specific focus on the dietary fiber pathway.

## Methods

### Research subjects

This study utilized data from the CLHLS from the 2008–2018 follow-up waves. CLHLS is a nationally representative, population-based longitudinal cohort study conducted across 23 provinces, municipalities, and autonomous regions in China. It employed a multi-stage, stratified cluster sampling method to enroll community-dwelling adults aged 65 years and older. Data on demographic characteristics, lifestyle factors, dietary habits, health status, and cognitive function were collected via face-to-face interviews. The validity and reliability of the CLHLS data have been well-documented in previous geriatric research (16).

Participants were included if they: (1) were aged ≥65 years at the baseline survey in 2008; (2) had completed the dietary and cognitive function assessments at baseline; (3) were free of dementia at baseline, as determined by the DSM-5 and ICD-10 diagnostic criteria; and (4) had complete follow-up data, with at least one cognitive assessment during the follow-up period. Participants were excluded if they: (1) had severe mental illness, malignant tumors, or other conditions at baseline that could potentially affect cognitive function; (2) had missing data on dietary intake or key covariates; or (3) were lost to follow-up or withdrew from the study for reasons other than dementia. A total of 1948 participants met the eligibility criteria and were included in the final analysis.

### Definition and measurement of key variables

#### Exposure factor: vegetarian diet intake

Dietary intake was assessed using items from the food frequency questionnaire (FFQ) included in the CLHLS survey, which covered 10 food categories: meat, fish, eggs, dairy products, legumes. Grains, vegetables, fruits, nuts, and fungi. For plant-based foods (legumes, grains, vegetables, fruits, nuts, and fungi), a score was assigned based on the frequency of consumption: 1 point for “rarely or almost never,” 2 points for “occasionally,” and 3 points for “weekly or daily.” These scores were summed to create a fiber-based vegetarian score, which ranged from 5 to 15. A meat food intake score was similarly constructed based on the consumption frequency of animal-derived foods (meat, fish, eggs, and dairy products), with total scores ranging from 4 to 12. (Note: Beans, as their main contribution is plant protein, have not been included in the fiber-based vegetarian score).

#### Outcome indicator: dementia

Follow-up assessments were conducted in 2010, 2011, 2014, and 2018 to identify incident dementia cases. Participants were followed until the diagnosis of dementia, death, or the end of the follow-up period, whichever occurred first. Dementia diagnosis was determined based on the following criteria: (1) Cognitive function assessed using the Chinese version of the Mini-Mental State Examination (CMMSE), with education-specific cutoffs. Specifically, participants with no formal education were classified as having cognitive decline if their CMMSE score was ≤16; those with 1–6 years of education were classified as having cognitive decline if their score was ≤19; and those with more than 6 years of education were classified as having cognitive decline if their score was ≤23. Participants with scores above these cutoffs were considered to have typical cognitive function (17). (2) Functional impairment, defined as a score of ≥7 on the Activities of Daily Living (ADL) scale; and (3) A self-reported physician diagnosis of dementia, based on an affirmative response to the question: “Have you ever been diagnosed with dementia by a hospital?” Participants or their proxies who answered “Yes” to this question were classified as having dementia. This composite diagnostic approach has been previously validated in CLHLS-based studies and demonstrates high reliability and validity for dementia ascertainment.

According to the Diagnostic and Statistical Manual of Mental Disorders (DSM-5) and the International Classification of Diseases, Tenth Edition (ICD-10), the diagnosis of dementia requires the simultaneous occurrence of cognitive and functional impairments, or a doctor’s confirmed diagnosis of dementia or related memory disorders. Thus, the three criteria (CMMSE, ADL, physician diagnosis) are not applied independently; instead, CMMSE and ADL must be satisfied simultaneously to meet the DSM-5/ICD-10 definition, while physician diagnosis provides an alternative independent basis for classification.

#### Covariate

Based on existing research and theoretical frameworks, the following potential confounding factors are adjusted: (1) Demographic characteristics: age (continuous variable), gender (male/female), education level, marital status, income, occupation; (2) Lifestyle factors: smoking status (Yes / No), drinking status (Yes / No), physicallabor (Yes / No); (3) Health status: chronic diseases such as hypertension, diabetes, heart disease, stroke (Yes / No), body mass index (BMI, continuous variable).

### Statistical analysis

Statistical analyses were performed using SPSS (version 26.0) and R software (version 4.3.0). Continuous variables with a normal distribution were presented as mean ± standard deviation, and between-group comparisons were conducted using Student’s *t*-tests. Non-normally distributed continuous variables were expressed as median (interquartile range), and group comparisons were performed using the Wilcoxon rank-sum test. Categorical variables were summarized as frequencies (percentages), and between-group differences were examined using chi-square (χ^2^) tests.

Cox proportional hazards regression models were employed to examine the association between vegetarian dietary intake and incident dementia. Hazard ratios (HRs) and their 95% confidence intervals (CIs) were calculated. Two models were constructed: Model 1 was unadjusted; Model 2 was adjusted for demographic characteristics (sex, age, cigarette, drinking, education, marital, ethnic, income, hypertension, diabetes, coronary heart disease, stroke, physicallabor, occupation).

Subgroup and sensitivity analyses were performed to test the robustness of our findings. These included: (1) excluding participants who developed dementia within the first 2 years of follow-up to minimize potential reverse causality; (2) excluding participants with age values in the top and bottom 5% of the distribution to mitigate the influence of extreme age; (3) Including legumes in the assessment, an expanded version of the fiber-based vegetarian score was constructed.; (4) In order to better discuss whether exclusion causes selection bias, we calculated propensity scores for the baseline characteristics (age, gender, chronic disease status) of the included participants. and (5) conducting stratified analyses by age (<80 vs. ≥80 years), sex, and education level to explore potential effect modification. All statistical tests were two-sided, and a *p*-value < 0.05 was considered statistically significant.

## Results

**Figure 1:**
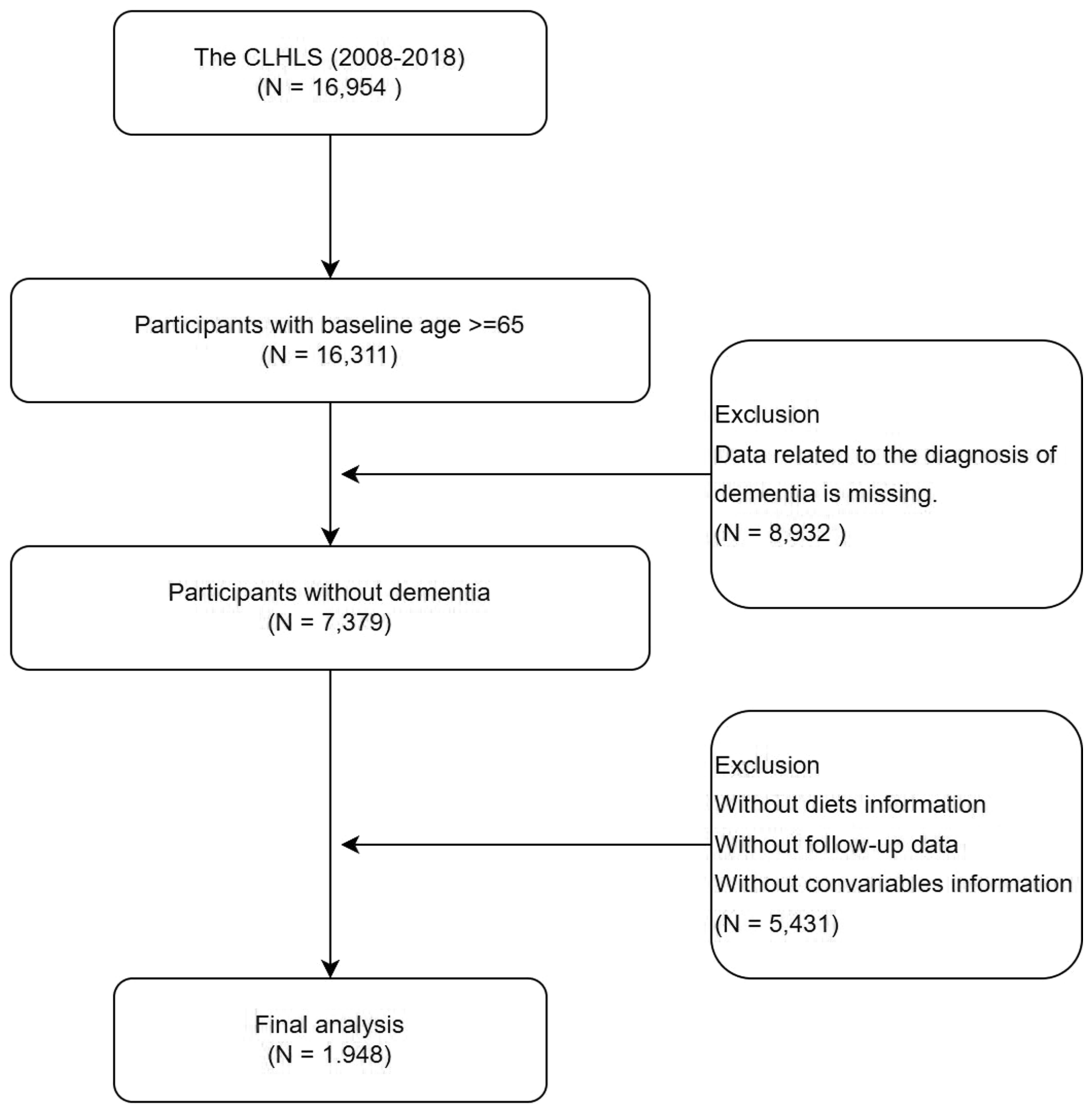
Flowchart.

We utilized the longitudinal data from the CLHLS waves from 2008 to 2018, which included 16,954 initial participants in 2008. After excluding 8,932 individuals who lacked data related to dementia, and subsequently eliminating cases with incomplete information on independent variables or covariates, the final analysis cohort consisted of a total of 1,948 individuals (see Figure 1).

### Baseline characteristics of the study population

This study included a total of 1,948 elderly individuals aged 65 and above from the CLHLS 2008 to 2018. Among them, 53 cases (2.7%) were diagnosed with dementia during the follow-up period. Table 1 shows the comparison of demographic characteristics and health conditions between the dementia group and the non-dementia group at baseline. Compared with the non-dementia group, the dementia group was older (80 years vs. 74 years, *p* < 0.001), might had a lower BMI (21.4 vs. 22.3, *p* = 0.099), a higher proportion of females (67.9% vs. 51.6%, *p* = 0.019), and there was a significant difference in marital status distribution (54.7% for widowed vs. 36.9%, *p* = 0.022). There were no statistically significant differences in education level, smoking, drinking, chronic disease history (hypertension, diabetes, heart disease, stroke), and physical labor between the two groups (*p* > 0.05).

**Table 1:** Participant demographics and baseline characteristics.

Characteristic	Dementia		*p*-value
	No *N* = 1,895	Yes *N* = 53	
Age, Median (IQR)	80 (74, 86)	74 (69, 80)	<0.001^1^
BMI, Median (IQR)	21.4 (19.2, 23.8)	22.3 (20.0, 24.5)	0.099^1^
*Ethnic, n (%)*			0.363^2^
Han	1,792 (94.6%)	52 (98.1%)	
Other	103 (5.4%)	1 (1.9%)	
Cigarette, *n* (%)	656 (34.6%)	17 (32.1%)	0.701^3^
Drinking, *n* (%)	502 (26.5%)	10 (18.9%)	0.214^3^
*Sex, n (%)*			0.019^3^
Female	977 (51.6%)	36 (67.9%)	
Male	918 (48.4%)	17 (32.1%)	
*Marital, n (%)*			0.022^2^
Currently married and living with spouse	1,112 (58.7%)	23 (43.4%)	
Divorced	5 (0.3%)	1 (1.9%)	
Never married	19 (1.0%)	0 (0.0%)	
Separated	59 (3.1%)	0 (0.0%)	
Widowed	700 (36.9%)	29 (54.7%)	
*Education, n (%)*			0.170^2^
Collage or above	114 (6.0%)	1 (1.9%)	
High school	89 (4.7%)	3 (5.7%)	
Middle school	133 (7.0%)	4 (7.5%)	
No education	895 (47.2%)	33 (62.3%)	
Primary school	131 (6.9%)	4 (7.5%)	
Primary school but not graduated	533 (28.1%)	8 (15.1%)	
Hypertension, *n* (%)	618 (32.6%)	15 (28.3%)	0.509^3^
Diabetes, *n* (%)	86 (4.5%)	4 (7.5%)	0.306^2^
Heart disease, *n* (%)	248 (13.1%)	9 (17.0%)	0.409^3^
Stroke, *n* (%)	127 (6.7%)	4 (7.5%)	0.778^2^
Physical labor, *n* (%)	702 (37.0%)	19 (35.8%)	0.859^3^
*Occupation, n (%)*			
Agriculture, forestry, animal husbandry or fishery worker	1,073 (56.6%)	35 (66.0%)	
Commercial, service or industrial worker	212 (11.2%)	3 (5.7%)	
Governmental, institutional or managerial personnel	63 (3.3%)	3 (5.7%)	
House worker	94 (5.0%)	5 (9.4%)	
Military personnel	15 (0.8%)	0 (0.0%)	
Never worked	7 (0.4%)	1 (1.9%)	
Others	322 (17.0%)	4 (7.5%)	
Professional and technical personnel	77 (4.1%)	2 (3.8%)	
Self-employed	32 (1.7%)	0 (0.0%)	
Income, Median (IQR)	6,000 (3,000, 50,000)	17,500 (5,000, 40,000)	0.166^1^

1 Wilcoxon rank sum test.

2 Fisher’s exact test.

3 Pearson’s Chi-squared test.

### Association between different food intake frequency and dementia

Table 2 shows the distribution of food intake frequencies in the dementia group and the non-dementia group. In terms of dairy product intake, the proportion of “usually or daily intake” in the dementia group was higher than that in the non-dementia group (37.7% vs. 23.3%), and the difference between the groups was close to the significance level (*p* = 0.060); the proportion of “usually or daily intake” of fungi foods in the dementia group was lower (5.7% vs. 17.3%), also close to significance (*p* = 0.080); in terms of nut intake, the proportion of “usually or daily intake” in the dementia group was 7.5%, which was lower than 16.6% in the non-dementia group (*p* = 0.211). There were no significant differences in the intake frequencies of the remaining food categories (meat, fish, eggs, grains, vegetables, fruits, and beans) between the two groups (all *p* > 0.05).

**Table 2:** Food intake frequency distribution among dementia and non-dementia groups.

Characteristic	Dementia		*p*-value
	No *N* = 1,895	Yes *N* = 53	
Meat, *n* (%)			0.067^1^
Occasionally	250 (13.2%)	4 (7.5%)	
Rarely or never	376 (19.8%)	17 (32.1%)	
Usually or everyday	1,269 (67.0%)	32 (60.4%)	
Fish, *n* (%)			0.996^1^
Occasionally	391 (20.6%)	11 (20.8%)	
Rarely or never	690 (36.4%)	19 (35.8%)	
Usually or everyday	814 (43.0%)	23 (43.4%)	
Eggs, *n* (%)			0.413^1^
Occasionally	230 (12.1%)	5 (9.4%)	
Rarely or never	360 (19.0%)	7 (13.2%)	
Usually or everyday	1,305 (68.9%)	41 (77.4%)	
Dairy, *n* (%)			0.060^2^
Occasionally	126 (6.6%)	2 (3.8%)	
Rarely or never	1,328 (70.1%)	31 (58.5%)	
Usually or everyday	441 (23.3%)	20 (37.7%)	
Grains, *n* (%)			0.338^1^
Occasionally	417 (22.0%)	13 (24.5%)	
Rarely or never	213 (11.2%)	9 (17.0%)	
Usually or everyday	1,265 (66.8%)	31 (58.5%)	
Vegetables, *n* (%)			0.158^2^
Occasionally	89 (4.7%)	4 (7.5%)	
Rarely or never	34 (1.8%)	2 (3.8%)	
Usually or everyday	1,772 (93.5%)	47 (88.7%)	
Fruits, *n* (%)			0.853^1^
Occasionally	637 (33.6%)	16 (30.2%)	
Rarely or never	455 (24.0%)	14 (26.4%)	
Usually or everyday	803 (42.4%)	23 (43.4%)	
Legumes, *n* (%)			0.835^1^
Occasionally	423 (22.3%)	10 (18.9%)	
Rarely or never	452 (23.9%)	13 (24.5%)	
Usually or everyday	1,020 (53.8%)	30 (56.6%)	
Nuts, *n* (%)			0.211^1^
Occasionally	242 (12.8%)	7 (13.2%)	
Rarely or never	1,339 (70.7%)	42 (79.2%)	
Usually or everyday	314 (16.6%)	4 (7.5%)	
Fungi, *n* (%)			0.080^1^
Occasionally	338 (17.8%)	10 (18.9%)	
Rarely or never	1,229 (64.9%)	40 (75.5%)	
Usually or everyday	328 (17.3%)	3 (5.7%)	

1 Pearson’s Chi-squared test.

2 Fisher’s exact test.

### Cox regression analysis of vegetarian diet pattern and risk of dementia

The association between vegetarian dietary patterns and dementia risk was analyzed using Cox proportional hazards regression models. The results are presented in Table 3. When the vegetarian dietary score was modeled as a continuous variable, each one-unit increase was associated with a 17% lower risk of dementia after full adjustment for covariates (HR = 0.83, 95% CI: 0.70–0.98, *p* = 0.031). Participants were categorized into tertiles based on their vegetarian dietary score (Vegetarianism T1, T2, and T3). Using the lowest tertile (T1) as the reference, participants in the middle tertile (T2) had a 51% lower risk of dementia (HR = 0.49, 95% CI: 0.26–0.93, *p* = 0.028), and those in the highest tertile (T3) had a 50% lower risk (HR = 0.50, 95% CI: 0.23–0.99, *p* = 0.048), indicating a significant protective effect. In contrast, no significant associations were observed for the meat intake score (T3 and T2 vs. T1) and the total dietary score (T3 and T2 vs. T1) in relation to dementia risk (*p* > 0.05). When a direct comparison was made between meat intake and total food intake, the results indicated that an increase in meat intake led to an increase in the risk of dementia (HR = 1.38, 95% CI: 1.10–1.74, *p* = 0.007). Additionally, an increase in total intake was associated with a reduced risk (HR = 0.81, 95% CI: 0.68–0.97, *p* = 0.019), but no significant statistical differences were observed in stratified comparisons. These findings suggest that a dietary pattern characterized by predominantly plant-based foods is associated with a reduced risk of dementia among Chinese older adults, and the results remained robust in adjusted analyses.

**Table 3:** Cox regression analysis of the association between different dietary intakes and the risk of dementia.

Characteristic	*N*	Event *N*	HR	95% CI	*p* value	aHR	95% CI	*p* value
Vegetarianism	1,948	53	0.79	0.67, 0.94	0.006	0.83	0.70,0.98	0.031
Meat	1,948	53	1.09	0.95, 1.24	0.221	1.38	1.10,1.74	0.007
Total	1,948	53	0.96	0.87,1.05	0.348	0.81	0.68,0.97	0.019
Vegetarianism 1	505	24	—	—	—	—	—	—
Vegetarianism 2	857	18	0.44	0.24,0.82	0.010	0.49	0.26,0.93	0.028
Vegetarianism 3	533	11	0.43	0.21,0.90	0.024	0.50	0.23,0.99	0.048
Meat 1	573	12	—	—	—	—	—	—
Meat 2	536	21	1.87	0.91,3.84	0.088	2.06	0.99,4.30	0.051
Meat 3	786	20	1.22	0.59,2.51	0.598	1.48	0.71,3.11	0.295
Total 1	540	19	—	—	—	—	—	—
Total 2	734	17	0.66	0.34,1.28	0.217	0.75	0.38,1.47	0.401
Total 3	621	17	0.78	0.40,1.51	0.459	0.92	0.48,1.93	0.915

CI, confidence interval; HR, hazard ratio, Adjusted: Sex, age, cigarette, drinking, education, marital, ethnic, income, hypertension, diabetes, heart disease, stroke, physical labor, occupation.

### Subgroup analysis

The protective effect of a vegetarian diet varies among different subgroups. Significant negative correlations were observed in participants younger than 80 years old (HR = 0.62, 95% CI: 0.48–0.80, *p* < 0.001), non-drinkers (HR = 0.81, 95% CI: 0.67–0.97, *p* = 0.021), those without formal education (HR = 0.79, 95% CI: 0.63–0.98, *p* = 0.033), those engaged in manual labor (HR = 0.66, 95% CI: 0.49–0.89, *p* = 0.006), and those who were married and lived with their spouses (HR = 0.70, 95% CI: 0.54–0.90, *p* = 0.006). No significant correlations were found in participants older than or equal to 80 years old, males, drinkers, those with any education, those not engaged in manual labor, or those with other marital statuses (*p* > 0.05) (see Table 4).

**Table 4:** Subgroup cox regression analysis of the association between vegetarian intake and the risk of dementia.

Characteristic	*N*	Event *N*	HR	95% CI	*p*-value
Sex					
Female	977	36	0.82	0.67, 1.00	0.054
Male	918	17	0.79	0.59, 1.06	0.122
Age					
Age < 80	1,353	25	0.62	0.48, 0.80	0.000
Age ≥ 80	542	28	1.05	0.84, 1.31	0.656
BMI					
BMI < 24	1,327	41	0.83	0.68, 1.00	0.050
BMI ≥ 24	568	12	0.75	0.53, 1.06	0.107
Cigarette					
No	1,239	36	0.82	0.67, 1.00	0.052
Yes	656	17	0.76	0.56, 1.04	0.090
Drinking					
No	1,393	43	0.81	0.67, 0.97	0.021
Yes	502	10	0.81	0.54, 1.20	0.288
Education					
No education	895	33	0.79	0.63, 0.98	0.033
Other	1,000	20	0.88	0.68, 1.14	0.333
Physical labor					
No	1,193	34	0.89	0.72, 1.11	0.300
Yes	702	19	0.66	0.49, 0.89	0.006
Marital					
Married, cohabiting	1,112	23	0.70	0.54, 0.90	0.006
Other	783	30	0.92	0.74, 1.15	0.451

CI, confidence interval; HR, hazard ratio.

### Sensitivity analysis

We performed four sensitivity analyses to assess the robustness of our findings. First, we excluded participants who were diagnosed with dementia within the first 2 years of follow-up. The association between vegetarian dietary intake and reduced dementia risk persisted (HR = 0.86, 95% CI: 0.72–0.93, *p* = 0.007). Second, given the significant age difference observed between the dementia and non-dementia groups at baseline, we excluded participants with age values in the top and bottom 5% of the distribution to further assess the stability of our results. This analysis confirmed that the protective effect of a vegetarian dietary pattern remained significant (HR = 0.81, 95% CI: 0.68–0.95, *p* = 0.011). In the sensitivity analysis using the expanded fiber-based vegetarian score that included legumes, the unadjusted model showed a significant association with lower dementia risk (HR = 0.87, 95% CI: 0.76–0.99, *p* = 0.038). However, after full adjustment for covariates, the association was no longer statistically significant (HR = 0.89, 95% CI: 0.78–1.02, *p* = 0.101). The baseline characteristics (age, gender, chronic disease status) of the included participants were used to calculate the propensity scores, and the subjects were weighted to make their distribution of key characteristics consistent with that of the dementia group. The Cox model was re-fitted, the association between the dietary fiber-based vegetarian score and the risk of dementia remained significant (HR = 0.63, 95% CI: 0.50–0.81, *p* < 0.001), which was consistent with the results of the main analysis.

## Discussion

This study, based on longitudinal data from the CLHLS (2008–2018), systematically evaluated the associations between the consumption frequency of 10 food groups, as well as overall vegetarian dietary patterns, and the risk of incident dementia. Cox proportional hazards regression revealed that, after multivariable adjustment, the vegetarian dietary pattern was significantly associated with a lower risk of dementia (HR = 0.83, 95% CI: 0.70–0.98, *p* = 0.031), suggesting that a dietary pattern characterized by predominantly plant-based foods may confer protective effects against cognitive decline. When participants were categorized into tertiles based on their vegetarian dietary score, those in the middle (T2) and highest (T3) tertiles exhibited a 51% (HR = 0.49) and 50% (HR = 0.50) lower risk of dementia, respectively, compared to the lowest tertile, indicating a more pronounced protective effect with greater adherence to a plant-based diet. In contrast, analyses of individual food groups revealed no significant associations between the consumption frequency of animal-based foods (e.g., meat, fish, eggs) and dementia risk, underscoring the importance of examining overall dietary patterns rather than isolated food components.

Based on this, our subgroup analysis revealed the significant heterogeneity of this protective effect among different demographic groups, providing more clinical evidence for understanding its mechanism of action and formulating precise nutritional intervention strategies. Firstly, the study found that the protective effect of a vegetarian diet was extremely significant in participants under the age of 80 (HR = 0.62), but the effect disappeared in the elderly group (≥80 years old). This result is largely consistent with the findings of Yang, that dietary intervention is more effective in people with intact cognitive function (18). From a pathophysiological perspective, the pre-clinical stage of dementia may last for several decades, and elderly people may be in the accumulation or irreversible stage of neurodegenerative changes. At this point, the scope for reversing cognitive decline through dietary adjustment is relatively limited. This suggests that a prevention strategy centered on vegetarianism may have an “opportunity window period”, and implementing it in middle-aged and elderly people may yield greater health benefits.

Secondly, the modifying effect of lifestyle factors warrants attention. The protective association of a plant-based dietary pattern was significant among non-drinkers (HR = 0.81, 95% CI: 0.67–0.97, *p* = 0.021), but not among drinkers. This finding may be attributable to the dual effects of alcohol on the nervous system: chronic alcohol consumption may not only directly induce neuronal damage but also interfere with the absorption and metabolism of key nutrients found in plant-based foods (e.g., B vitamins and folate), thereby potentially attenuating the beneficial effects of a plant-based dietary pattern (19, 20). These observations suggest that promoting a plant-based dietary pattern alongside recommendations to reduce or avoid alcohol intake may may confer a synergistic benefit; however, this hypothesis arises from observational subgroup findings and requires testing in future interventional or well-controlled prospective studies.

Of particular interest, the protective association of a vegetarian diet pattern was most pronounced among participants with no formal education (HR = 0.79, 95% CI: 0.63–0.98, *p* = 0.033) and those engaged in manual labor (HR = 0.66, 95% CI: 0.49–0.89, *p* = 0.006). This finding carries important public health implications. On one hand, individuals with no formal education and those engaged in manual labor are more likely to be socioeconomically disadvantaged. Their habitual dietary patterns may be characterized by a higher intake of refined carbohydrates and high-salt, high-fat foods, with a correspondingly lower consumption of healthful plant-based foods (16, 21, 22). Consequently, adopting a plant-based dietary pattern rich in dietary fiber, vitamins, and antioxidants (e.g., from vegetables, fruits, and fungi) may yield a greater marginal benefit in terms of nutritional improvement, thereby conferring a more substantial cognitive protective effect. On the other hand, manual labor itself may increase energy expenditure and elevate oxidative stress levels. The abundant antioxidants inherent in vegetarian diets may precisely counteract this oxidative burden, thereby establishing a tightly coupled protective mechanism at the “demand–supply” interface.

Finally, the analysis of social support factors—specifically marital status—revealed that the protective association of a vegetarian diet pattern was significant only among participants who were married and living with their spouse. This finding may reflect the socially embedded nature of dietary behaviors: cohabitation with a spouse may facilitate the formation and maintenance of regular, healthier family eating habits, thereby enhancing adherence to and the potential benefits of a vegetarian diet pattern.

### Comparison with previous studies

The findings of the present study are consistent with and extend a series of recent investigations utilizing CLHLS data. Xian et al. (15). constructed plant-based diet indices (overall PDI, hPDI, and uPDI) using cross-sectional data from the 2018 CLHLS and reported that each one-unit increase in the healthful plant-based diet index (hPDI) was associated with a 2.4% lower odds of dementia (OR = 0.976, 95% CI: 0.963–0.990), whereas the unhealthful plant-based diet index (uPDI) was positively associated with dementia odds (OR = 1.012, 95% CI: 1.001–1.024). Similarly, a cross-sectional study by Zhang et al. (23)., also using 2018 CLHLS data, demonstrated that each one-point increment in the Chinese version of the MIND (cMIND) dietary score was associated with an 11% reduction in the odds of dementia (OR = 0.89, 95% CI: 0.84–0.93), with evidence of a linear dose–response relationship. These two cross-sectional studies provided important contextual support for the present investigation; however, their inherent design limitations precluded causal inference, as they could only identify associations rather than establish temporal sequences. The longitudinal design of the current study strengthens the evidence for a potential causal relationship between plant-based dietary patterns and dementia risk.

The findings of the present study are also consistent with multiple cohort studies conducted in broader international settings. de Crom et al. (24), using data from the Rotterdam Study cohort in the Netherlands, reported that a healthful plant-based diet index was inversely associated with dementia risk (HR = 0.86, 95% CI: 0.75–0.99). Morris pioneered the MIND dietary pattern, demonstrating a significant protective effect against Alzheimer’s disease risk in Western populations (HR = 0.47, 95% CI: 0.26–0.86) (25). Wu analyzed UK Biobank data and observed that hPDI was inversely associated with dementia risk, whereas uPDI was positively associated; however, the overall PDI showed no significant association (26). The latest meta-analysis on hPDI has found that hPDI is associated with a reduced risk of dementia by integrating plant-based diets from multiple countries (27). These research results are in line with the significant but single-food insignificant results in this study, further suggesting that attention should be paid to the overall dietary pattern rather than isolated nutrients.

### Potential biological mechanisms

The protective effects of vegetarian diet against dementia are biologically plausible and may involve multiple hypothesized pathways. It is important to note that our study did not directly measure these biological mechanisms; therefore, the following discussion is speculative and intended to generate hypotheses for future research. First, vegetarian foods are abundant in dietary fiber, vitamins (including folate, vitamin C, and vitamin E), minerals, and polyphenolic compounds (e.g., flavonoids and anthocyanins), which potentially potent antioxidant and anti-inflammatory properties. These bioactive compounds could attenuate neuroinflammatory responses and oxidative stress-induced damage, thereby potentially preserving neuronal structure and function (28, 29). Second, vegetarian diet patterns are typically associated with lower intakes of total energy, saturated fatty acids, and cholesterol, which might help maintain healthy vascular endothelial function and reduce the risk of cardiovascular conditions such as hypertension and atherosclerosis. Cerebrovascular health is, in turn, a critical foundation for preserving cognitive function (30, 31). Third, vegetarian diets may modulate the composition of the gut microbiota and promote the production of metabolites such as short-chain fatty acids. These metabolites could influence neurotransmitter synthesis, synaptic plasticity, and neuroinflammatory responses via the gut-brain axis (32). Fourth, specific food constituents might exert direct neuroprotective effects. For instance, fungi are rich in ergothioneine and *β*-glucan, compounds known for their antioxidant and anti-inflammatory properties (33); nuts provide unsaturated fatty acids and vitamin E, which may enhance cerebrovascular function; and tea contains catechins capable of crossing the blood–brain barrier and inhibiting β-amyloid aggregation (17). These proposed mechanisms, while supported by prior experimental studies, remain hypothetical in the context of our observational findings and require confirmation through future mechanistic studies.

### Advantages and limitations

This study possesses several strengths. First, the present study employed a large-sample, prospective cohort design with a follow-up period of up to 10 years, which strengthens the temporal basis for causal inference. Second, multivariable adjustment and stratified analyses were performed to account for major confounding factors, and sensitivity analyses were conducted to mitigate potential reverse causality, yielding relatively robust findings. Third, the dietary assessment encompassed 10 major food categories and enabled the construction of a comprehensive plant-based dietary score, offering a more holistic approach than analyses of individual food items.

Nevertheless, several limitations of this study should be acknowledged. First, dietary data were derived from self-reported food frequency questionnaires, which are subject to recall bias and measurement error. Additionally, the lack of information on cooking methods and total energy intake may have affected the precision of effect estimates. Second, the ascertainment of dementia was primarily based on cognitive function scores and assessments of daily living abilities. Although education-adjusted cutoffs were applied, the absence of a clinical gold standard for diagnosis (e.g., neuroimaging or biomarkers) may have resulted in some degree of misclassification bias. Third, despite controlling for numerous potential confounders, the possibility of residual confounding due to unmeasured factors (e.g., genetic susceptibility, micronutrient intake, social engagement) cannot be entirely ruled out. Fourth, the relatively small number of incident dementia cases (*N* = 53) may have limited our statistical power to conduct in-depth analyses of interaction effects and dose–response relationships across specific food categories. Fifth, the study population was restricted to older adults in China; therefore, caution is warranted when extrapolating these findings to other ethnic or age groups. Although we conducted a sensitivity analysis incorporating legumes into the vegetarian score, the null finding after adjustment may reflect residual confounding or limited statistical power rather than a true absence of effect. Future studies with larger sample sizes are needed to clarify the role of legumes in plant-based dietary patterns for dementia prevention. In this study, the CLHLS FFQ covers only 10 food categories, omitting potentially important dietary confounders such as tea, coffee, processed foods, and refined carbohydrates. This limits our ability to adjust for these factors, and residual confounding cannot be ruled out. Future studies with more detailed dietary assessments should validate our findings. We strongly recommend that the findings of this study be verified in future prospective cohorts with larger sample sizes and more sufficient event numbers (such as combining multiple waves of the CLHLS or conducting a meta-analysis). Finally, this score was established for the first time in the CLHLS population in this study and has not yet undergone external validation in other independent cohorts; future research should evaluate its reliability and validity.

## Conclusion

In conclusion, this study demonstrated that a diet mainly consisting of vegetarian foods was significantly associated with a lower risk of dementia in elderly Chinese individuals, especially in those with a moderate and high level of vegetarianism.

## Data Availability

The datasets presented in this study can be found in online repositories. The names of the repository/repositories and accession number(s) can be found in the article/supplementary material.
